# Editorial: Non-pharmacological interventions in healthy and pathological aging: Facts and perspectives

**DOI:** 10.3389/fnagi.2023.1191281

**Published:** 2023-04-18

**Authors:** Danúbia de Sá-Caputo, Adérito Seixas, Redha Taiar, Eddy A. Van der Zee, Mario Bernardo-Filho

**Affiliations:** ^1^Laboratório de Vibrações Mecânicas e Práticas Integrativas (LAVIMPI), Departamento de Biofísica e Biometria, Instituto de Biologia Roberto Alcantara Gomes and Policlínica Universitária Piquet Carneiro, Universidade do Estado do Rio de Janeiro, Rio de Janeiro, Brazil; ^2^Programa de Pós-Graduação em Fisiopatologia Clínica e Experimental, Faculdade de Ciências Médicas, Universidade do Estado do Rio de Janeiro, Rio de Janeiro, Brazil; ^3^Escola Superior de Saúde Fernando Pessoa, Fundação Fernando Pessoa, Porto, Portugal; ^4^MATériaux et Ingénierie Mécanique (MATIM), Université de Reims Champagne- Ardenne, Reims, France; ^5^Department of Molecular Neurobiology, Groningen Institute for Evolutionary Life Sciences (GELIFES), University of Groningen, Groningen, Netherlands

**Keywords:** aging, non-pharmacological intervention, cognitive function, physical exercise, mental health

## Introduction

The elderly population is defined as people aged 60 years and older according to the World Health Organization. The concept “aging population” is, from a historical point of view, a contemporary issue. The number of older people has been increasing, mainly in developing countries. There is data reporting that in 2019 this number was 1 billion and probably will increase to 1.4 billion in 2030 and even 2.1 billion in 2050, considering the world population. In fact, in 2019 only 9% of the world population was aged ≥65 years but the figures are expected to increase to 16% by 2050 (United Nations, 2019; Rudnicka et al., 2020; OECD, 2023).

Aging is a physiological phenomenon which can be related to healthy or pathological processes. The decline of physical and mental conditions, related to locomotor, cognition, and bodily functions, is associated with frailty syndrome and, consequently, with mortality. To the development of health aging, i.e., developing and maintaining the functional ability that enables wellbeing in older age, it is necessary to establish healthy habits, considering physical exercise, diet, mental health, quality of sleep, and other approaches throughout the life cycle. The environment in which people live can influence health and, consequently, the aging process. Cognitive functions and mood can be negatively affected during aging, increasing the risk of development of depression, dementia, and the deterioration of brain functions due to neurodegenerative diseases. Other health conditions are more prevalent in older people, such as obesity, benign prostatic hyperplasia, cardiovascular disease, stroke, and urinary incontinence (Ayensa and Calderon, 2011; Araujo et al., 2014; Salthouse, 2019; Badal et al., 2020; Bae, 2021; Arnoldy et al., 2023; Kim et al., 2023; Liu et al., 2023).

Therefore, strategies that promote healthy aging and thereby preventing impairments in quality of life, are important. The use of non-pharmacological interventions in the management of physical and/or mental impairments is desirable as they can be considered minimally invasive, effective and, generally, have are low-cost. Consequently, a better understanding of the mechanisms involved in these approaches is crucial (Sá-Caputo et al., 2014; Chen et al., 2015; Biagi et al., 2016; Conelea et al., 2017; Bennett et al., 2019; Boehme et al., 2021; Arauz et al., 2022; Cardoso et al., 2022; Tseng et al., 2023).

In this context, research involving health strategies such as dietary interventions, physical exercise, and cognitive exercise is needed as these interventions are expected to improve muscle strength, functionality, quality of life, quality of sleep, cognitive function and to help manage phenomena as depression, cardiovascular-, and urinary-related conditions, among other common health issues in older people. New approaches as whole-body vibration, fecal microbiota transplantation and transcranial magnetic stimulation have been reported as important strategies to prevent and manage health conditions in older people. Good adherence, easy application, reduced adverse effects and costs are reported advantages of these non-pharmacological interventions (Basso et al., 2019; Gaitán et al., 2020; Dal Farra et al., 2021; Liu et al., 2022; Nawrat-Szołtysik et al., 2022). However, non-pharmacological interventions serving health strategies are still understudied.

The aim of this Research Topic was to publish original papers and reviews describing the mechanisms related to the use of non-pharmacological interventions to prevent and to manage health conditions through the life cycle. Moreover, we focused on the presentation of data that can develop a better understanding regarding neuroscience aspects related to it, promoting evidence-based clinical practice.

In this special issue, ten articles addressing those questions are included. We summarize their major contributions according to the subject categories. One Brief Research, seven Original Research Papers, one Study Protocol and one Systematic Review. The *Brief Research* reported the similarities and differences regarding the antidepressant effect of repetitive transcranial magnetic stimulation in younger and older adults (Cotovio et al.). Seven *Original Research Papers* reported the effects of sulforaphane intake on processing speed and negative moods in healthy older adults (Nouchi et al.), the association between social engagement and depressive symptoms in middle-aged and elderly people (Yang et al.), the association of sleep quality with lower urinary tract symptoms/benign prostatic hyperplasia (Li et al.), the effect of regular fecal microbiota transplantation and the effect of whole-body vibration as a passive alternative to exercise after myocardial damage in middle-aged in mice (Zhang et al.), the effects of a specific Tai Chi concept on trunk postural control after stroke (Cui et al.), and the effects of a multidisciplinary body weight reduction program on physical and mental health and fatiguability of older people with obesity (Usubini et al.). The *Study Protocol* of a trial aimed to assess the effects of a 6-month multi-domain exercise program combining multiple exercise modalities, meditation, and social interaction on memory and brain function, in cognitively healthy late middle-aged and older adults (Chang et al.). The *Systematic Review* compared the efficacy and acceptability of treatments for depressive symptoms in people with cognitive impairment (Jin et al.). Taken these studies together it is made clear that progress is being made and new avenues lie ahead of us in the use of non-pharmacological interventions.

## Conclusion

As the number of people with old age has increased worldwide, the promotion of knowledge and strategies to achieve healthy aging is desirable and necessary. Considering the impact of health conditions during the aging process, approaches with a minimum of side effects, with good adherence, and low cost are needed to manage the negative impact on health and to promote healthy aging. Thus, research on the effectiveness of non-pharmacological interventions is relevant and increasingly important, given the urgency of increasing numbers of older people worldwide. Non-pharmacological approaches reported in this Research Topic, such as “repetitive transcranial magnetic stimulation”, “sulforaphane intake”, “physical exercise”, “meditation”, “social interaction”, “fecal microbiota transplantation”, “whole-body vibration”, “physiological treatments”, “music therapy”, “Tai Chi”, “body weight reduction programs”, “rehabilitation programs”, and “dietary interventions” seem to support improvements in cognitive, mental and physical function and, therefore, promote healthy aging and manage the consequences related to pathological aging.

## Author contributions

All authors listed have made a substantial, direct, and intellectual contribution to the work and approved it for publication.
